# Study protocol for factors influencing the adoption of ChatGPT technology by startups: Perceptions and attitudes of entrepreneurs

**DOI:** 10.1371/journal.pone.0298427

**Published:** 2024-02-15

**Authors:** Varun Gupta, Hongji Yang

**Affiliations:** 1 School of Computing and Mathematical Sciences, Leicester University, Leicester, England; 2 Multidisciplinary Research Centre for Innovations in SMEs (MrciS), Gisma University of Applied Sciences, Potsdam, Germany; 3 Department of Economics and Business Administration, University of Alcala, Alcalá de Henares (Madrid), Madrid, Spain; Sri Eshwar College of Engineering, INDIA

## Abstract

**Background:**

Generative Artificial Intelligence (AI) technology, for instance Chat Generative Pre-trained Transformer (ChatGPT), is continuously evolving, and its userbase is growing. These technologies are now being experimented by the businesses to leverage their potential and minimise their risks in business operations. The continuous adoption of the emerging Generative AI technologies will help startups gain more and more experience with adoptions, helping them to leverage continuously evolving technological innovation landscape. However, there is a dearth of prior research on ChatGPT adoption in the startup context, especially from Entrepreneur perspective, highlights the urgent need for a thorough investigation to identify the variables influencing this technological adoption. The primary objective of this study is to ascertain the factors that impact the uptake of ChatGPT technology by startups, anticipate their influence on the triumph of companies, and offer pragmatic suggestions for various stakeholders, including entrepreneurs, and policymakers.

**Method and analysis:**

This study attempts to explore the variables impacting startups’ adoption of ChatGPT technology, with an emphasis on comprehending entrepreneurs’ attitudes and perspectives. To identify and then empirically validate the Generative AI technology adoption framework, the study uses a two-stage methodology that includes experience-based research, and survey research. The research method design is descriptive and Correlational design. Stage one of the research study is descriptive and involves adding practical insights, and real-world context to the model by drawing from the professional consulting experiences of the researchers with the SMEs. The outcome of this stage is the adoption model (*also called as research framework*), building Upon Technology Adoption Model (TAM), that highlight the technology adoption factors (*also called as latent variables*) connected with subset of each other and finally to the technology adoption factor (or otherwise). Further, the latent variables and their relationships with other latent variables as graphically highlighted by the adoption model will be translated into the structured questionnaire. Stage two involves survey based research. In this stage, structured questionnaire is tested with small group of entrepreneurs (*who has provided informed* consent) and finally to be distributed among startup founders to further validate the relationships between these factors and the level of influence individual factors have on overall technology adoption. Partial Least Squares Structural Equation Modeling (PLS-SEM) will be used to analyze the gathered data. This multifaceted approach allows for a comprehensive analysis of the adoption process, with an emphasis on understanding, describing, and correlating the key elements at play.

**Discussion:**

This is the first study to investigate the factors that impact the adoption of Generative AI, for instance ChatGPT technology by startups from the Entrepreneurs perspectives. The study’s findings will give Entrepreneurs, Policymakers, technology providers, researchers, and Institutions offering support for entrepreneurs like Academia, Incubators and Accelerators, University libraries, public libraries, chambers of commerce, and foreign embassies important new information that will help them better understand the factors that encourage and hinder ChatGPT adoption. This will allow them to make well-informed strategic decisions about how to apply and use this technology in startup settings thereby improving their services for businesses.

## Introduction

### Background

Artificial intelligence (AI) has advanced tremendously and revolutionized several industries. AI driven applications has the functional capabilities to generate new content, graphics, audio, and video, is called generative artificial intelligence (AI) [1]. Generative AI are one recent applications of AI which learn from large datasets (*training datasets)* and then produce unique and innovative content human reply style for the users in response to their queries (*also called prompts*) [2]. Generative Pre-trained Transformer (ChatGPT) is one example of a generative AI model that has been trained on vast quantities of data to generate textual responses in a chat-like format in response to user inquiry. The continuous research by OpenAI is being conducted to continuously innovate ChatGPT, for instance launch of new multimodal large language model called GPT-4 compared to previous GPT-3.5. The latest model is reported to have better capabilities to provide more accurate responses, better problem solving, and better features, for instance images analysis rather just text analysis (https://openai.com/gpt-4). Innovations in generative AI technology are expected to unfold rapidly, intensifying competition among industry players to develop better generative AI technologies. The market is poised to witness a continuous influx of innovative Generative AI technologies, leading to the emergence of sophisticated applications that either incorporate inbuilt generative AI or are purposefully built using such technologies. These innovations will create great value for its users, for instance, entrepreneurs, who will have the opportunity to navigate the rapidly evolving landscape of such technologies to harness its potential benefits for their ventures. Given that AI is still in its early stages of development, they must also take these challenges into account. The integration of these technologies with business practices seems not to be a *future of work problem which is too distant*; it is going to happen soon; at least looking at the pace of innovations in Generative AI technologies.

Numerous businesses had been actively using ChatGPT, and it was stated that their use had a positive relationship with the productivity [3], and success of the company [4]. The ChatGPT quality, Information quality, and Service quality is reported to have positive impact on user benefits and their satisfaction. This ultimately positively impact the organizational performance [3]. For instance, the system quality, information quality and service quality factors can result in accurate and timely market knowledge for the marketing team, thereby enhancing the user benefits their satisfaction levels. This may result in the better marketing decisions, for instance, better pricing decisions, and better impact, for instance, higher sales and business performance. Additionally, the positive impacts of adoption of ChatGPT includes cost reduction and operational efficiency [5, 6] support for Business Model Innovations [7], and possibility of integration in one of the crucial areas of Research and Innovation [8]. Although this technology is still in its early phases, it appears to be worthwhile for businesses, including startups to investigate further. Startups employ a range of effortful methods to perform market research, expert and customer interviews, internet research tools, and analysis of secondary materials, for instance, published market reports. For startups with limited resources, ChatGPT may be a useful knowledge collaborator. It can assist the startup team in utilizing the information generated by this technology to augment the data obtained from their current market research procedures. More thorough data collecting, and analysis could help improve the multiple points of view that were produced, which would result in rational business model innovations.

The literature has examined the adoption of AI technology by either extending theoretical models, such as the Unified Theory of Acceptance and Use of Technology (UTAT) model, and Technology Acceptance Model (TAM) [9], or by proposing new models, such as the Task-oriented AI Acceptance (T-AIA) model [10] and Artificially Intelligent Device Use Acceptance model (AIDUA) [11]. Nonetheless, research focused solely on the acceptance of generative AI technologies—such as ChatGPT—has examined the acceptance from the perspectives of Students [12–16], knowledge workers [17], healthcare professionals [18], and customers [19]. To undertake such investigations, the researchers has extended TAM model [12–15], UTAT Model [16, 17], and AIDUA Model [19] as the research frameworks. The dearth of prior research on ChatGPT adoption in the startup context, especially from Entrepreneur perspective, highlights the urgent need for a thorough investigation to identify the variables influencing this technological adoption. Determining the factors that contribute to a startup’s successful integration of ChatGPT is critical because they operate under unique working contexts and constraints. This study investigates these characteristics to give entrepreneurs critical insights that will help them make rational decisions, reduce technology related risks having a business impact, and foster their continuous innovations in their entrepreneurial environment. With time, more advanced and innovative Generative AI technologies and applications based on them will hit the market as the AI field develops. The continuous adoption of the emerging Generative AI technologies will help startups gain more and more experience with adoptions, helping them to leverage continuously evolving technological innovation landscape.

### Rationale

It is crucial to comprehend the factors that affect startups’ adoption of ChatGPT technology. This will enable the entrepreneurs to make decisions on whether they should adopt the technology. For instance, the results will require startups to consider if their working environment and available resources match the requirements for a successful adoption. Additionally, the results will allow them to decide whether to execute the small-scale, incremental experiments that should be the foundation for adopting AI technology [20, 21]. For example, the entrepreneurs will be able to design small experiments based on the adoption factors and accurately identify the performance criteria for these experimentations. The results have implications for different stakeholders, for instance, Entrepreneurs, Policymakers, technology providers, researchers, and Institutions offering support for entrepreneurs like Academia, Incubators and Accelerators, University libraries, public libraries, chambers of commerce, and foreign embassies. These stakeholders will find the research findings useful in improving their understanding of the adoption determinants and obstacles, which will improve their ability to make decisions about their service innovations and, ultimately improve the environment for entrepreneurship.

### Objectives

The primary objective of this study is to ascertain the factors that impact the uptake of ChatGPT technology by startups, anticipate their influence on the triumph of companies, and offer pragmatic suggestions for various stakeholders, including entrepreneurs, and policymakers.

## Research design and methodology

The research design employed for this study encompasses descriptive and correlational research designs. The understanding built upon a review of the literature’s existing adoption models for AI technologies in general and for generative AI adoption models in particular, will be refined further with the experience-based research (*researcher’s real-world experiences with SMEs in supporting their business operations and Digital Transformations*). *Stage one* of the research study is descriptive and involves adding practical insights, and real-world context to the adoption model. This descriptive research involved experience research to build the model’s which is having sufficient informative depth and comprehensiveness and provides a more thorough knowledge of the phenomenon being studied (*the model which is otherwise missing in the literature*).

The technology adoption model will be made up of various factors (*also called as latent variables) that will be connected to other latent variables*. Each connection is a hypothesis that need to be empirically tested later research stages by employing a survey-based approach. After the empirical validation of the model, the validity of the impact of one latent variable on another and ultimately on overall technology adoption (*or otherwise*) could be established. *Stage two* is purely correlational research with the focus on exploring relationships between various factors (*or latent variables*) associated with the adoption of Generative AI technology among startup entrepreneurs (*model created in Stage One*). In this stage, the data is collected from the entrepreneurs and analysed using Partial Least Squares Structural Equation Modeling (PLS-SEM) approach without any manipulation of the independent variable leading to the statistical analysis of the relationships between the adoption model latent variable (*or factors*).

The research methodology consists of a two-stage approach (***Two-Stage Approach*):**

- **Stage One (Research Framework formulation):** Literature lacks the Generative AI technology adoption model pertaining to the startups and based on the perspectives of the Entrepreneurs. Nevertheless, the literature has examined the adoption of AI technology in service industry and had proposed new adoption models, for instance, Task-oriented AI Acceptance (T-AIA) model [10] and Artificially Intelligent Device Use Acceptance model (AIDUA) [11]. Researchers has also investigated the acceptance of generative AI technologies—such as ChatGPT—by extending TAM model [12–15], UTAT Model [16, 17], and AIDUA Model [19] as the research frameworks. The technology adoptions were empirically investigated from the perspectives of Students [12–16], knowledge workers [17], healthcare professionals [18], and customers [19]. The literature lacks studies that are conducted in context of Startups from the perspectives of the Entrepreneurs.

These models, especially, AIDUA and T-AIA do provide useful factors that result in adoption of AI technologies but need to be further refined to align it with the specifics of Generative AI technologies, specifics of startups unique characteristics, and from the perspectives of the Entrepreneurs. The TAM model (TAM) describes how people come to accept and use new technologies and provides the criteria for technology adoption. [22, 23] but need to be modified further as for intelligent AI systems, its prediction power is not higher [11]. Stage one of the research project will modify the TAM model by incorporating new factors based on the professional experiences of researchers, and the study of current models for AI adoption, such as the Task-oriented AI Acceptance (T-AIA) model [10], and artificially intelligent (AIDUA) device use acceptance (model) [11], to make it better aligned for Generative AI technology adoption prediction.

With an array of consulting experience, researchers have provided active support to small and medium-sized businesses (SMEs) in a variety of their business operations in both domestic and international markets, for instance, providing valuable information to foster business model innovations. Additionally, support had been provided in the smooth integration of AI technologies like ChatGPT, for digitally transformation of business operations. The researcher’s consulting support has been valuable for these SMES in innovating their business models and facilitating rapid scaling in competitive markets. The SMEs also benefited from consulting support for the adoption of AI technology, which led to the technology’s active use in business operations and the teams’ constant experimentation with new technologies. Additionally, the professional connections with SMEs that are not currently utilizing generative AI technologies but have expressed a desire to do so in the future, are beneficial to identify the attributes that may facilitate their adoption in such cases. These experiences, which span the AI adoption spectrum and include SMEs with varying AI technology adoption experiences and life cycle stages, are valuable sources of knowledge for drafting Generative AI technology adoption frameworks. By leveraging valuable insights from these cases, the technology adoption framework will be driven by more practical insights and aligned with SMEs characteristics. Additionally, there had been continuous discussions with other professionals and entrepreneurs about technology adoption. The valuable insights from those conducted discussions at workplace, workshops, and other business meetings, will be useful in formulating the technology adoption model (*research framework*). Further, the latent variables and their relationships with other latent variables as graphically highlighted by the adoption model will be translated into the structured questionnaire. This Research framework then will be empirically validated in *Stage two* of the research project, which is survey based, to validate the relationships between these factors and the level of influence individual factors have on overall technology adoption.

- **Stage Two (Survey-Based Approach):** The research uses a survey-based approach. Startups will be requested to take part in the study by providing them with participant information sheet, General Data Protection Regulation (GDPR) privacy notice, and Informed consent form to fill (*initial request) (See Sampling Subsection of Data Management and Analysis section)*. Thereafter, after receiving the informed consent form from the entrepreneurs who agree to take part in the research study, the questionnaire as prepared in *Stage* one, will be finalised after conducting the pilot testing with a small group of these participants. This will help to identify any ambiguities or issues with survey questions, ensuring clarity and relevance. The final questionnaire will then be distributed to the participants. The collected data will then be statistically analysed leading to the empirical testing of the technology adoption framework *(See data analysis Subsection of Data Management and Analysis section)*. This questionnaire will include factors identified in Stage One (*research framework)* that impact the adoption of ChatGPT technology among entrepreneurs. To give a thorough grasp of the adoption process, more data about startup working context, industry context, and past experiences with AI technology will also be gathered (*if the participants provide consent for sharing them with research team and hence the research audience)*.

### National and local approvals/ethical considerations and approval

The research study is approved by the Institutional Review Board of Winning Scientific Management, Lisbon, Portugal under protocol number 0823J. The research project is also approved by the University of Leicester Ethics Sub-Committee of Science & Engineering, Arts, Humanities and Law (*research study design ID*: *43400*). The research project has also obtained clearance under the ***Academic Technology Approval Scheme (ATAS)*** of the UK Government’s Foreign, Commonwealth, and Development Office (FCDO) dated July 20th, 2023.

### Data analysis

Partial Least Squares Structural Equation Modeling (PLS-SEM) will be used to analyze the gathered data. PLS-SEM offers a powerful statistical analytic tool to ascertain the relevance and influence of each factor on startups’ adoption of ChatGPT technology, as well as the ability to examine complicated interactions among variables. The study will analyze the structural connections among the suggested constructs, appraise the importance of these connections, and ascertain the degree of their influence. PLS-SEM is selected for the data analysis because this technique is reported to work well for small sample sizes (*Entrepreneurs using ChatGPT for business operations are increasing but still comprises small population size)*, complex models (*adoption model is complex with diverse factors and interactions)* and does not make any assumptions about data distribution (*not sure if Likert scale data collected in the research will be normal or otherwise) [24, 25]*. Further, this technique is well suited if the objective of the research is to develop the theory, explain how much of the variability in your dependent variables (constructs) can be accounted for by the independent variables (factors or predictors) in your model, and predicting the relationships between different constructs within the adoption theoretical model [25].

### Follow-up

A procedure for follow-up will be instituted to guarantee that the experiences and concerns of the participants are considered. During the *Stage two* of the research study, during the filling of the survey questionnaire, participants will be given the opportunity to indicate their willingness to engage in follow-up interviews, for instance, exploring reasons behind analysed statistical correlations in research framework or other inquiries, for instance, seeking their general opinion about future implications, by providing their contact information in the questionnaire itself. The goal of these optional follow-up conversations is to gather rich perspectives about the analysed results, especially unexpected outcome situations (*if any*). This will enable researchers to offer more in-depth explanations of the elements influencing technology adoption (*just like case study explanations)* that simply survey-based research would not be able to answer (*quantitative based)*. The follow-ups are totally optional, and it is possible to participate in the research without sharing any personal information. Interviews will be scheduled at the convenience of follow-up participants, who will only be contacted by phone, email, or online meetings if that is their chosen method of communication. Researchers will have a deeper grasp of the multiple viewpoints about the research problem through multiple rounds of follow-ups. This will contribute to extending the research study’s outcomes and enhancing its reliability and validity.

## Data management and analysis

### Sampling

Purposive or convenience sampling, a non-probability sample technique, will be employed in the study, which will feature startups from the researcher’s professional network (*and extended network)* in universities, public libraries, accelerators, incubators, and similar institutions. The researcher intends to focus on startups in their professional network (*and extended* network) that are either considering adopting ChatGPT technology or have rich experiences or prior experience with it. These startups will be picked because they can offer insightful information on the factors driving technology adoption and because they are in line with the goals of the research (*they have experience with ChatGPT technology adoption)*.

ChatGPT is recently introduced in the market and its integration with the business practices is still under active experimentation by the entrepreneurs. Entrepreneurs maybe using ChatGPT in their business operations at varying degrees, for instance, for easy task of drafting marketing messages to knowledge intensive task of market research. Its use is now less widespread, but it is growing as more peer success stories are made public and entrepreneurs are scaling their experimentations with the technology. The population size of entrepreneurs actively using ChatGPT for business operations seems to be smaller but cannot be estimated quantitatively. Assuming the confidence level of 95% and ±5% of the margin errors, the sample size is computed to be 385.

The startups in researchers’ professional network as well in the extended professional network, will receive an invitation to participate along with information on the goals, nature of the research project, and eligibility criteria for participation (*active use or having used Generative AI in their business operations*). The research project information, for instance, research objectives, voluntary participation, associated risks and benefits, data confidentiality, specifics about personal data collection (if any), safety precautions (*refer to the Ethics and Safety Considerations section*), and contact persons, will be shared with potential participants to help them make an informed decision about whether or not to take part in the research. The University of Leicester’s standard participant information sheet (*annexure-A attached in S1 Annex*) will be used to disseminate research project details. The participant information sheet will provide participants with sufficient project-related information to enable them to make an informed decision about their participation in the project. The Participants will also be shared the General Data Protection Regulation (GDPR) privacy notice which applies if they wish to share their personal data. The University of Leicester’s GDPR Privacy Notice explains how it gathers and utilizes participants’ personal data when they participate in the research project (*annexure-B attached in S1 Annex*). Participants agreeing to participate will provide their written informed consent using the University of Leicester’s standard Informed consent form (*annexure-C attached in S1 Annex)*. After receiving consent, the researcher will give the participating startups structured questionnaires to complete to gather data. The founders of the startup have to option to explicitly give their permission to be contacted in future for necessary follow-ups (*refer to the data collection section)* by providing their personal information (*refer to data storage section for information about retention period)*. In case they expressed their consent to participate in the follow-up sessions, new participant information sheet (*annexure-D attached in S1 Annex*), and informed consent form will be shared (*annexure-E attached in S1 Annex*). The conditions expressed before the start of the survey, for instance data processing, ethical requirements, GDPR notice, will all remain the same for the follow-up sessions.

### Data collection

The startup entrepreneurs will be given a standardized questionnaire (*which is built based on outcomes of stage one of the research study)* to complete to gather data. The informed consent will be taken before distributing the questionnaire (*refer to the Ethics and Safety Considerations section)*. The questionnaire will be created through Google Forms and distributed among the research study participants. To guarantee precision and effectiveness, the answers will be electronically recorded (*Google spreadsheet)*. The questionnaire will also have the fields where participants could enter their organization details, their contact details (*in case interested in follow-ups)*, background knowledge of AI technology, industry context, specific challenges, and success stories about their firms. The survey form will not record any personal information unless and until the participant explicitly feeds the information for follow-ups. This means that even the e-mail ids won’t be recorded but they will have the option to edit their response after submission (*they just need to save their survey link with the option “Edit your response”)*.

### Data storage

An electronic database that is password-protected and safe will house all the gathered data. This database will only be accessible to members of the authorized research team. The EU and UK GDPR laws does not specify the time period for which personal data can be retained. Businesses are free to choose how long to keep personal information, for instance, based on the purpose it was collected. The stored personal data will be deleted 6 months after the end of the research study to allow ample time for any follow-ups and research article drafting. *The collected data (5-point Likert scale values) as well as personal data (if shared by entrepreneurs) will not be shared with any third parties*, *even outside EEA*. *The 5-point Likert scale research data will also be destroyed beyond final publication (publication of Stage two results*).

### Data analysis

The Partial Least Squares Structural Equation Modeling (PLS-SEM) method will be used to conduct a thorough analysis of the gathered survey data. This approach will evaluate the correlations between the variables, determine the relevance of the results, and quantify the magnitude of the effects. For this, the SmartPLS 3.2.9 software version will be used.

### Data handling procedures

Data management will adhere to ethical and privacy standards. The research team leaders will be the only ones with access to the data, as it won’t be available to other parties. Individual data gathered from the survey will not be shared in any follow-up correspondence with respondents. Additionally, the volunteer researchers will only be able to access the 5-point Likert scale values that have been registered for each question in the questionnaire (*removing any personal or corporate-specific information*) to guarantee that analytic methods are carried out appropriately (*refer to the Quality Assurance section)*. Only when participants voluntarily provide their personal information, it will be gathered (for follow-ups). Specifically, it is feasible to participate in a research study without disclosing any personal information or information unique to the participating business. All submitted information will only be used in aggregate form; no personal information will be disclosed by the research project. Only with consent from the participants (*captured in informed consent form*) will the specific quotes that they contributed to the questionnaire or in the follow-ups be made public (*but anonymised*, *no personal information will be revealed*).

## Quality assurance

Periodic quality control checks will be carried out to find and fix any flaws or discrepancies in the data. To improve the quality of the data gathered, data cleaning and validation will be done. The participants (*those who gave consent when completing the questionnaire*, *refer to the data collection section*) will be contacted in the follow-ups to seek their opinions on the findings and to confirm the overall conclusions of the study. Additionally, the volunteer researchers will replicate the analysis on a dataset to verify the accuracy of the execution of the analysis processes (*personal or company-specific information will be eliminated*, *and only 5-point Likert scale values will be retained*, *refer to data handling procedure section*). The survey questionnaire will only be finalised after a small pilot test conducted with the group of entrepreneurs (*only those that had submitted informed consent forms)* to ensure that it is non-ambiguous, complete, and clear for the participants. The questionnaire will have clear instructions to participants to ensure that all the respondents are aware of way survey form is to be filled. Finally, involvement of both researchers in all stages of research will help them to cross-check each other work (*peer-review)*, reducing individual bias through discussions and deliberations on various research aspects, and collaborative problem solving by integrating diverse perspectives.

## Problems anticipated

The study may encounter several hurdles or impediments, such as participant dropout, non-responses to the survey, and potential issues in recruiting entrepreneurs. The research team will use techniques to successfully engage participants to overcome these problems, including transparent communication about the research, research implications, and ethical considerations of the study. This will be achieved by sharing detailed information through participant information sheet and General Data Protection Regulation (GDPR) privacy notice. There is a provision for follow-us (*only with the participants that agree by sharing their personal information)*, for instance queries after data analysis or sharing interesting outcomes of the research. This will keep participants engaged and motivated to contribute to the project. Further, to accommodate the busy schedules of entrepreneurs, they are offered full flexibility to fill the survey within 7 days either in one go (*filling the entire form altogether)* or incrementally (*they must submit the form and fill the pending fields at later time through “Edit your response option”)*. The google form won’t record the email Ids of the participants. Ideally the form filling is expected to take 30 mins time. The busy entrepreneurs opting for incremental filling of the form will have to spend on average 4 minutes per day to complete the form (*within a week deadline*). This will be helping entrepreneurs to contribute rather dropping out in the middle because they will have flexibility to fill the survey, reduced time pressure, and enough time to consider their responses thoughtfully. The survey form will be created in a user friendly manner but won’t have the option to send reminders (*as personal data won’t be collected*). The researchers will sample the startups from their professional networks, for instance at public libraries, universities, accelerators, incubators, etc (*refer to the sampling section)*.

## Expected outcomes and implications

The results of this study will add to the corpus of knowledge by identifying the variables influencing startups’ adoption of ChatGPT technology. The study’s findings will give Entrepreneurs, Policymakers, technology providers, researchers, and Institutions offering support for entrepreneurs like Academia, Incubators and Accelerators, University libraries, public libraries, chambers of commerce, and foreign embassies important new information that will help them better understand the factors that encourage and hinder ChatGPT adoption. This will allow them to make well-informed strategic decisions about how to apply and use this technology in startup settings thereby improving their services for businesses. As listed below, the research effort has an array of outcomes and implications for important stakeholders of the research project.

- **Entrepreneurs**

**Outcomes:** Business owners will get a better comprehension of the factors influencing ChatGPT adoption. They will have access to a framework for the adoption of technology, perceptions of possible obstacles, and factors to consider when making technology adoption decisions.

**Implications:** By using the research results, entrepreneurs may evaluate if ChatGPT technology is feasible for their ventures, determine effective adoption strategies, and possibly improve their creativity and competitiveness by optimal decision-making about the adoption of suitable AI technology. For instance, if system quality plays a significant role in deciding whether or not to implement Generative AI, then they can adopt the solution that is reported to have the best quality, for instance, accuracy, amongst multiple competing brands.

- **Policymakers**

**Outcomes:** Research results will inform the policymakers to help them make well-informed regulatory decisions to better help the startup community utilize cutting-edge technologies. The factors, and hindrances as reported by the research will help policymakers to adapt regulations and/or come up with assistance services for the startup community, for instance, legal assistance or a better innovation ecosystem.

**Implications:** This research can assist decision-makers in formulating laws that encourage innovation, encourage technology adoption in startups, and establish conducive environments for the expansion of entrepreneurship, for instance by providing better support to the startup community. Legislators could create research centers to support businesses in need of assistance if, for instance, adopting Generative AI technology requires a thorough evaluation of system quality and these startups lack the necessary competence.

- **Technology Providers**

**Outcomes:** Technology companies will learn more about the unique requirements, difficulties, and preferences of startup customers, which will help them with improvisation in the technology and better customization for the startups.

**Implications:** Technology suppliers can better support startups and possibly grow their client base by customizing their solutions based on their understanding of the factors influencing adoption. For instance, if the entrepreneurs are motivated to experiment with generative AI to gain a deeper understanding of its functional capabilities due in large part to perceived ease of use, technology providers can build the solution in a way that is easy for users to interact with, thereby improving their overall experience.

- **Researchers**

**Outcomes:** By adding to the body of knowledge already available on technology adoption and entrepreneurship, researchers may open the door to future research possibilities and scholarly publications.

**Implications:** This study can contribute to our knowledge of technology adoption and lay the groundwork for more research in this area in the future. For instance, further research could foster innovations in Generative AIs that are more aligned with the startup needs and working context.

- **Institutions Offering Support for Entrepreneurs (e.g., Academia, Incubators, Accelerators)**

**Outcomes:** Organizations offering support for startups will find research results valuable to improve their programs for entrepreneurship. For instance, they will better understand how they can further enhance their problem-solving skills by strategic integration of the technology and by leveraging across full functional capabilities of it could provide better support to the startups. They could also transfer their experiences with these technologies to the startups thereby helping them to expand their capabilities by adopting these technologies seamlessly.

**Implications:** Organizations can more effectively enhance their competencies to better offer business support to the startups, for instance, market research. They can also help startups to enhance their dynamic capabilities to solve their business problems by better adoption of the technology.

- **University Libraries and Public Libraries**

**Outcomes:** By including research findings about the adoption of technology in startups, libraries can broaden their offerings in terms of resources and services. For instance, this will help libraries to improve their literature review services [26], and global market research support [27, 28].

**Implications:** By making this study results available to the libraries, they can keep informed about current trends in the AI domain and help them make well-informed technology adoption decisions. This will foster digital transformations in libraries, helping them innovate their services for traditional patrons and entrepreneurs. For instance, the adoption framework, which is the research project’s output, is also applicable to librarians. This will guarantee the thoughtful implementation of beneficial Generative AI technologies in libraries, as well as beneficial assistance from the innovation ecosystem to accomplish the adoption, such as from technology suppliers and policymakers.

- **Chambers of Commerce**

**Outcomes:** The Chamber of Commerce is an association composed of businesses and business professionals within a specific geographic area, for instance, a city, with the primary objective of promoting the interests of the local business community. Research results will help the members of the association to learn about the patterns of technology adoption, thereby helping them make strategic adoption decisions that help them solve their business problems. This also helps them to support the other members in various business support programs, for instance, training programs, market research support, etc.

**Implications:** By using this information, chambers of commerce can provide networking and support opportunities that strengthen links and promote growth within the local business community. Additionally, by exchanging best practices, the network might accelerate adoption factors, which would ultimately result in the acceptance of useful technologies and the rejection of less valuable ones.

- **Foreign Embassies**

**Outcomes:** The research results will help foreign embassies to boost their commercial diplomacy by innovating their services for supporting the globalization of their home country businesses [29]. This could mean that the embassies can leverage across full functional capabilities of the technology to provide meaningful business information to the startups.

**Consequences:** Should embassies aid startups overseas, the study can assist in customizing support and advice to correspond with the unique obstacles and prospects encountered by global business owners. The adoption framework, which is the research project’s output, is also applicable to the foreign embassies. This model will provide useful directions for the successful adoption of valuable Generative AI technologies in embassy departments offering business support, as well as beneficial assistance from the innovation ecosystem to accomplish the adoption, such as from technology suppliers and policymakers.

## Result dissemination

The findings of this study will be published in high-quality and indexed international journals. The research study’s first-stage results will be published as a short article or commentary in a journal of practice. The research study’s second-stage results will be published as a full research article in the Journal of Practice. Additionally, the participants as well as relevant stakeholders will have access to a detailed report that summarizes the research findings, implications, and suggestions.

## Ethics and safety considerations

### Informed consent

Informed consent will be obtained from research participants before starting data collection. They will receive complete disclosures about the objectives of the study, the research methods to be employed, the non-disclosure of the results, the privacy of their personal information, and how the research study will use their answers. This will be accomplished by sharing participant information sheet, General Data Protection Regulation (GDPR) privacy notice, and Informed consent form to fill. Before taking part, they will have the chance to ask questions (*through email)* and give their voluntary consent (*through signed informed consents)*. In any event, participants are free to leave the research project at any moment and without giving a reason.

### Privacy and confidentiality

To preserve participant privacy, personal information will only be collected (*for follow-ups*) when participants willingly submit it. The researchers will have no ways for requesting the participants for their personal information (*as the option will only be available in survey form and it is impossible to reach out back to them as their emails will not be recorded)*. It is possible to take part in a research study without giving out any private information or information exclusive to the participating company. No personal information will be revealed by the study project (*if shared by participants)*; all provided data (*Likert scale values*) will only be utilized in aggregate form. No personal information will be disclosed, for instance in publications, without the consent of the participant. The participants’ approval will be obtained before making public the precise quotes they provided at the time of filling the questionnaire. The research data (Likert scale values and personal information) will only be accessible to leaders of the research team while volunteer researchers (*if any*) will only have access to 5-point Likert scale values (*research data excluding personal information*). The personal or company-specific information will be eliminated from the dataset shared with the volunteer researchers.

### Minimizing harm

Participants can complete the questionnaire without difficulty or stress because it can be completed in a week at their own pace (*possibility of completing in increments in a week*). There is no risk of physical injury because the entire process can be completed online from the comfort of the participant’s home or place of business.

### Data security

The safety and privacy of participant information will be guaranteed by the implementation of data security measures, such as password protection and secure storage. Only the research project leaders will have access to the storage. To guarantee that no unauthorized individual can access the research data, the workstation used to access it will be password protected. In case the research data do not include any personal information then it will be simply stored on the university cloud storage through OneDrive file hosting service. The OneDrive allows the data to be shared with the peer researcher for research collaboration. It is not possible to access the data without an official login and granting access to the research data. In case personal data is also collected (*in case participants shared personal data for follow-ups)*, the University’s Records Manager will be requested to add it to the official register as per the Regulations the University. To prevent sharing of this data on third-party storage, it will only be shared within the researcher’s password-protected computer memory.

## Supporting information

S1 Annex (DOCX)
