# Supplementary material for: Study protocol for factors influencing the adoption of ChatGPT technology by startups: Perceptions and attitudes of entrepreneurs
Source: PLoS One. 2024 Feb 15;19(2):e0298427. doi: 10.1371/journal.pone.0298427 (PMC10868733; doi:10.1371/journal.pone.0298427)
Supplement: S1 Annex — (DOCX) [file pone.0298427.s001.docx]

**Annexure-A**

**University of Leicester's standard participant information sheet**

# Research Project title

Factors Influencing the Adoption of ChatGPT Technology by Startups: Perceptions and Attitudes of Entrepreneurs

# Invitation paragraph

### You are being invited to take part in a research project. Before you decide whether or not to take part, it is important for you to understand why the research is being done and what it will involve. Please take time to read the following information carefully'.

# What is the purpose of the research project?

The primary objective of this study is to ascertain the variables that impact the uptake of ChatGPT technology by startups, anticipate their influence on the triumph of companies, and offer pragmatic suggestions for various stakeholders, including entrepreneurs, and policymakers.

# Why have I been invited to participate?

Based on your active integration of ChatGPT in your business operations, your experience will provide great value to the research. This research will then have a great implication for the business community. By using the research results, entrepreneurs may evaluate if ChatGPT technology is feasible for their ventures, determine effective adoption strategies, and possibly improve their creativity and competitiveness by optimal decision-making about the adoption of suitable AI technology.

# Do I have to take part?

It is up to you to decide whether or not to take part in this research project. If you do decide to take part, you will be given this information sheet along with a privacy notice that will explain how your data will be collected and used and be asked to provide your consent to participate. If you decide to take part you are still free to withdraw at any time and without giving a reason, by contacting the researcher.

# What will happen to me if I take part?

The startup entrepreneurs will be given a standardized questionnaire (which is built based on outcomes of stage one of the research study) to complete to gather data. The informed consent will be taken before distributing the questionnaire. The questionnaire will be created through Google Forms and distributed among the research study participants. To guarantee precision and effectiveness, the answers will be electronically recorded (Google spreadsheet). The questionnaire will also have the fields where participants could enter their organization details, their contact details (in case they are interested in follow-ups), background knowledge of AI technology, industry context, specific challenges, and success stories about their firms. After receiving consent, the researcher will give the participating startups structured questionnaires to complete to gather data.

# What will happen to any samples that I provide?

**Sampling:** Purposive or convenience sampling, a non-probability sample technique, will be employed in the study, which will feature startups from the researcher's professional network in universities, public libraries, accelerators, incubators, and similar institutions. The researcher intends to focus on startups in their professional network that are either considering adopting ChatGPT technology or have prior experience with it. These startups will be picked because they can offer insightful information on the factors driving technology adoption and because they are in line with the goals of the research (they have experience with ChatGPT technology adoption). The qualified startups will receive an invitation to participate along with information on the goals and nature of the research project. Participants agreeing to participate will receive an informed consent statement detailing the study's objectives, methods, possible risks, and safety precautions. After receiving consent, the researcher will give the participating startups structured questionnaires to complete to gather data. If the founders of the startup have given their permission (and personal data), necessary follow-ups will be carried out.

**Data Collection:** The startup entrepreneurs will be given a standardized questionnaire (which is built based on outcomes of stage one of the research study) to complete to gather data. The informed consent will be taken before distributing the questionnaire. The questionnaire will be created through Google Forms and distributed among the research study participants. To guarantee precision and effectiveness, the answers will be electronically recorded (Google spreadsheet). The questionnaire will also have the fields where participants could enter their organization details, their contact details (in case interested in follow-ups), background knowledge of AI technology, industry context, specific challenges, and success stories about their firms.

**Data Storage:** An electronic database that is password-protected and safe will house all the gathered data. This database will only be accessible to members of the authorized research team. The stored data will be deleted 6 months after the publication of the research results.

# What are the possible disadvantages and risks of taking part? (where appropriate)

The study has no involved risks. 30 minute time is estimated for filling the survey form. You have 7 days’ time to fill it. This means that you can save your progress and return back to the form as when you want.

# What are the possible benefits of taking part?

**Outcomes:** Business owners will get a better comprehension of the factors influencing ChatGPT adoption. They will have access to a framework for the adoption of technology, perceptions of possible obstacles, and factors to consider when making technology adoption decisions.

**Implications:** By using the research results, entrepreneurs may evaluate if ChatGPT technology is feasible for their ventures, determine effective adoption strategies, and possibly improve their creativity and competitiveness by optimal decision-making about the adoption of suitable AI technology.

# What data will you collect about me?

We just need quantitative data from you (5-point Likert scale values). Providing personal data is optional and will be used for possible follow-ups in future.

# Will what I say in this research project be kept confidential?

Data management will adhere to ethical and privacy standards. The research team leaders will be the only ones with access to the data, as it won't be available to other parties. Individual data gathered from the survey will not be shared in any follow-up correspondence with respondents. Additionally, the volunteer researchers will only be able to access the 5-point Likert scale values that have been registered for each question in the questionnaire (removing any personal or corporate-specific information) to guarantee that analytic methods are carried out appropriately. Only the aggregated values of the individual responses will be published.

# How will you look after the data you collect about me?

### *We need to ensure that you understand what will happen to data we collect about you as well as your legal rights. This document is accompanied with a separate Privacy Notice providing further details. The copy is attached.*

### *Your normal rights under the Data Protection Act and the General Data Protection Regulation apply. However, we need to manage your records in specific ways for the research project to be reliable. This means that we won’t [always] be able to let you see or change the data we hold about you.*

### *You can stop being part of the research project at any time, without giving a reason, but we will keep information about you that we already have and continue to use this for the purposes of the research project as outlined here.* At all times this research study will comply with the UK General Data Protection Regulations (2018).

# What will happen to the results of the research project?

The results will be published in leading Journals. The personal information won’t be published except the cases where individuals explicitly make a request, for instance publication of the quotes.

# What should I do if I want to take part?

*You will be asked to complete an Informed Consent Form and to opt-in to a variety of research options by ticking the Yes or No box. This will confirm you understand how your data will be processed, protected, and reviewed for research purposes’.*

# Who is organising and funding the research project?

The research is joint research between Multidisciplinary Research Centre for Innovations in SMEs (MrciS), Gisma University of Applied Sciences, 14469 Potsdam, Germany and University of Leicester, UK. The research is funded by Winning Scientific Management, Portugal.

# What if something goes wrong?

*In the very unlikely event of you being harmed by taking part in this research project, there are no special compensation arrangements. If you are harmed due to someone’s negligence, then you may have grounds for legal action but you may have to pay for it.*

# Who has reviewed the research project?

The IRB approval is obtained from Winning Scientific Management, Portugal and University of Leicester, UK. Please contact Prof. Dr. Dr. Varun Gupta at vg109@leicester.ac.uk or Prof. Hongji Yang at [hongji.yang@leicester.ac.uk](mailto:hongji.yang@leicester.ac.uk), for any further information about research.

If you have any concerns or queries about the way in which the research project has been conducted, you should contact the Chair of the University Research Ethics Committee on [ethics@le.ac.uk](mailto:ethics@le.ac.uk).

*If you require more GDPR data protection information, then you can access this via the University’s Information Assurance Services:*

*Information Assurance Services*
*University of Leicester*
*University Road*
*Leicester*
*LE1 7RH*
*T: +44 (0)116 229 7945*
*E: dpo@le.ac*

*W:* <https://www2.le.ac.uk/offices/ias>’

### Thanks a lot for spending time in reading this information.

**Annexure-B**

**General Data Protection Regulation (GDPR) privacy notice**

# Privacy Notice for Research Participants

## Research Study title & Researcher Name

### Factors Influencing the Adoption of ChatGPT Technology by Startups: Perceptions and Attitudes of Entrepreneurs by Varun Gupta & Hongji Yang.

This Privacy Notice provides information about how the University of Leicester collects and uses your personal information when you take part in this research projects.

Please also refer to the Participant Information Sheet given to you for further details about the research project, what information will be collected about you, and how it will be used.

**The University of Leicester** will usually be the *Data Controller* of any data that you supply for this research. This means that we are responsible for looking after your information and using it properly. This means that the University will make the decisions on how your data is used and for what reasons. The exception to this is joint research projects, if this is applicable you will be informed on the Participant Information Sheet as to the other partner institution(s) who will also have responsibilities for looking after your information. You can access more information on this via the University’s Information Assurance Services:

Information Assurance Services
University of Leicester
University Road
Leicester
LE1 7RH
T: +44 (0)116 229 7945
E: [ias@le.ac.uk](mailto:ias@le.ac.uk)

W: <https://www2.le.ac.uk/offices/ias>

## Why do we need your data?

Sharing personal data is not mandatory. The personal data is only required for possible future follow-ups for instance, asking further questions after data collection.

**University of Leicester’s legal basis for collecting this data is:**

Processing is necessary for the performance of a task in the public interest such as research.

## What type of data will the University of Leicester use?

Only the quantitative data expressed as 5-point Likert scale values and personal data (if explicitly shared by the participants).

## Who will the University of Leicester share your data with?

The data will not be shared with any institution. Only the aggregated results will be published in leading Journals and then shared with the audience.

## Will the University of Leicester transfer my data outside of the UK?

No.

## What rights do I have regarding my data held by the University of Leicester?

Your normal rights under the Data Protection Act and the General Data Protection Regulation apply. However, we need to manage your records in specific ways for the research project to be reliable. This means that we will not [always] be able to let you see or change the data we hold about you.

You can stop being part of the research project at any time, without giving a reason, but we will keep information about you that we already have and continue to use this for the purposes of the research project as outlined in the Participant Information Sheet.

## Where did the University of Leicester source my data from?

Data will be collected from Entrepreneurs through survey forms.

## Are there any consequences of not providing the requested data?

There are no consequences of not providing data for this research. It is purely voluntary.

## Will there be any automated decision making using my data?

There will be no use of automated decision making in scope of UK Data Protection and Privacy legislation.

## How long will the University of Leicester keep my data?

In line with the law, we will only keep your data for as long as we need to so that we can fulfil our research objectives.

*We will keep your personal data (if shared) for maximum period of 6 months from when the study ends so as to successfully complete entire research (including follow-ups) and possible publication thereafter.*

## Who can I contact if I have concerns?

In the event of any questions about the research project, please contact the researchers in the first instance. Please contact Prof. Dr. Dr. Varun Gupta at [vg109@leicester.ac.uk](mailto:vg109@leicester.ac.uk) or Prof. Hongji Yang at [hongji.yang@leicester.ac.uk](mailto:hongji.yang@leicester.ac.uk)]

If you have any concerns about the way in which the research project has been conducted, please contact the **Chair of the** **University Research Ethics Committee at** [ethics@leicester.ac.uk](mailto:ethics@leicester.ac.uk).

The University of Leicester Data Protection Officer is:

*Data Protection Officer*

*University of Leicester,*

*University Road, Leicester, LE1 7RH*

*0116 229 7640*

*DPO@le.ac.uk*

For further details about information security, please contact the [**Information Assurance Services**](https://www2.le.ac.uk/offices/ias) team.

**Annexure-C**

**University of Leicester's standard Informed consent form**

**CONSENT FORM**

**Full title of Project:** Factors Influencing the Adoption of ChatGPT Technology by Startups: Perceptions and Attitudes of Entrepreneurs

**Name, position and contact details of Researcher:** Varun Gupta, Academic Visitor, vg109@leicester.ac.uk

**Name, position and contact details for Supervisor:** Hongji Yang, Professor, hongji.yang@leicester.ac.uk

|  | Please **initial** box | |
| --- | --- | --- |
| 1. I confirm that I have read and understand the participant information sheet for the above study and have had the opportunity to ask questions. |  | |
| 1. I understand that my participation is voluntary and that I am free to withdraw at any time, without giving reason. |  | |
| 1. I understand that at all times this research project will comply with the *General Data Protection Regulations (GDPR, 2018)* approved by the EU parliament on 14 April 2016 and passing into UK law effective from 25 May 2018 and that if I have any concerns how I contact the University of Leicester to raise these.   4. I agree to take part in the above research project. |  | |
|  | Please **initial** box | |
| 1. I agree to the use of anonymised quotes in publications. | Yes | No |
| 1. I agree that anonymised information, gathered about me for this research project may be stored in a specialist data centre/repository relevant to this subject area for future research. **The database will only be accessible to the lead researchers.** 2. I agree to being named in subsequent publications and understand this will be for academic purposes and not commercial gain. 3. I agree that data collected for this research project may be used in future research. 4. I wish to receive a copy of the results of this research project, and I agree for my contact details to be retained and used for this purpose. |  |  |

Name of Participant Date Signature

Name of Researcher obtaining informed consent Date Signature

**Annexure-D**

**University of Leicester's standard participant information sheet (***For Follow-ups****)***

# Research Project title

Factors Influencing the Adoption of ChatGPT Technology by Startups: Perceptions and Attitudes of Entrepreneurs

# Invitation paragraph

### *You are being invited to take part in a follow-up phase of the research project. We thank you for agreeing to take part in the follow-up (by recording your consent at the time of filling the survey form and sharing communication details*). However, you still can decide *whether or not to take part in the follow up, it is important for you to understand what will be involved in such follow-ups. Please take time to read the following information carefully'.*

# What is the purpose of the research project?

The primary objective of this study is to ascertain the variables that impact the uptake of ChatGPT technology by startups, anticipate their influence on the triumph of companies, and offer pragmatic suggestions for various stakeholders, including entrepreneurs, and policymakers.

# Why have I been invited to participate?

Many thanks for your active contribution in filling out the survey form. Your contribution is valuable for the research project that aims to investigate the factors that impact the adoption of Generative AI technologies, for instance, ChatGPT technology by the entrepreneurs in their startups. We have now analysed the results and would like to share some interesting results with you to gather your opinions about the same. The goal of these optional follow-up conversations is to gather rich perspectives about the analysed results, especially unexpected outcome situations (if any). This will enable researchers to offer more in-depth explanations of the elements influencing technology adoption (just like case study explanations) that simply survey-based research would not be able to answer (quantitative based).

# Do I have to take part?

It is up to you to decide whether or not to take part in this follow-up phase of the project. If you decide to take part, you will be asked for the communication channel convenient to you (*limited to telephone, emails, and online platforms for arranging online meeting).* If you decide to take part you are still free to withdraw at any time and without giving a reason, by contacting the researcher. The interview will be arranged in the best possible time convenient to you and the research team. The Privacy rules will remain the same as shared during pre-survey time period.

# What will happen to me if I take part?

Interviews will be scheduled at the convenience of follow-up participants, who will only be contacted by phone, email, or online meetings if that is their chosen method of communication. Researchers will have a deeper grasp of the multiple viewpoints about the research problem through multiple rounds of follow-ups. This will contribute to extending the research study's outcomes and enhancing its reliability and validity.

# What will happen to any samples that I provide?

Your responses will be investigated to better understand the results from different perspectives. This information will be really valuable to provide explanation about the results and other interesting facts about the research problem which cannot be captured otherwise in statistical analysis.

# What are the possible disadvantages and risks of taking part? (where appropriate)

The interview will last maximum 30 minutes. We don’t anticipate any risks involved in the follow-ups.

# What are the possible benefits of taking part?

Participation in the follow-ups will help research team understand better the research results and other themes that are not reflected by the results. This will help to provide not only empirical evaluation of the Generative AI adoption factors but also informative description and explanation of the phenomenon being investigated.

# What data will you collect about me?

We just need qualitative data from you, that will be driven from your opinions and experiences. No personal data will be captured. Only the anonymised quotes will be published.

# Will what I say in this research project be kept confidential?

Data management will adhere to ethical and privacy standards. The research team leaders will be the only ones with access to the data, as it won't be available to other parties. Individual data gathered from the survey will not be shared in any follow-up correspondence with respondents. Additionally, the volunteer researchers will only be able to access the 5-point Likert scale values that have been registered for each question in the questionnaire (removing any personal or corporate-specific information) to guarantee that analytic methods are carried out appropriately. The follow-up data will only be used to make the results more informative without making a reference to the source of the information. Only the aggregated values of the individual responses and the anonymised quotes will be published.

# How will you look after the data you collect about me?

### We need to ensure that you understand what will happen to data we collect about you as well as your legal rights. This document is accompanied with a separate Privacy Notice providing further details. The copy is attached. Your normal rights under the Data Protection Act and the General Data Protection Regulation apply. However, we need to manage your records in specific ways for the research project to be reliable. This means that we won’t [always] be able to let you see or change the data we hold about you. You can stop being part of the research project at any time, without giving a reason, but we will keep information about you that we already have and continue to use this for the purposes of the research project as outlined here. At all times this research study will comply with the UK General Data Protection Regulations (2018).

# What will happen to the results of the research project?

The results will be published in leading Journals. The personal information won’t be published except the cases where individuals explicitly makes a request, for instance publication of the quotes.

# What should I do if I want to take part?

*You will be asked to complete another Informed Consent Form and to opt-in to a variety of research options by ticking the Yes or No box. This will confirm you understand how your data will be processed, protected and reviewed for research purposes’.*

# Who is organising and funding the research project?

The research is joint research between Multidisciplinary Research Centre for Innovations in SMEs (MrciS), Gisma University of Applied Sciences, 14469 Potsdam, Germany and University of Leicester, UK. The research is funded by Winning Scientific Management, Portugal.

# What if something goes wrong?

*In the very unlikely event of you being harmed by taking part in this research project, there are no special compensation arrangements. If you are harmed due to someone’s negligence, then you may have grounds for legal action but you may have to pay for it.*

# Who has reviewed the research project?

The IRB approval is obtained from Winning Scientific Management, Portugal and University of Leicester, UK.

Please contact Prof. Dr. Dr. Varun Gupta at vg109@leicester.ac.uk or Prof. Hongji Yang at [hongji.yang@leicester.ac.uk](mailto:hongji.yang@leicester.ac.uk), for any further information about research.

If you have any concerns or queries about the way in which the research project has been conducted, you should contact the Chair of the University Research Ethics Committee on [ethics@le.ac.uk](mailto:ethics@le.ac.uk).

*If you require more GDPR data protection information then you can access this via the University’s Information Assurance Services:*

*Information Assurance Services*
*University of Leicester*
*University Road*
*Leicester*
*LE1 7RH*
*T: +44 (0)116 229 7945*
*E: dpo@le.ac*

*W:* <https://www2.le.ac.uk/offices/ias>’

### Thanks a lot for spending time in reading this information.

**Annexure-E**

**University of Leicester's standard Informed consent form (***For Follow-ups***)**

**CONSENT FORM** (*for follow-ups)*

**Full title of Project:** Factors Influencing the Adoption of ChatGPT Technology by Startups: Perceptions and Attitudes of Entrepreneurs

**Name, position and contact details of Researcher:** Varun Gupta, Academic Visitor, vg109@leicester.ac.uk

**Name, position and contact details for Supervisor:** Hongji Yang, Professor, hongji.yang@leicester.ac.uk

|  | Please **initial** box | |
| --- | --- | --- |
| 1. I confirm that I have read and understand the participant information sheet for the above study (*for follow-ups)* and have had the opportunity to ask questions. |  | |
| 1. I understand that my participation is voluntary and that I am free to withdraw at any time, without giving reason. |  | |
| 1. I understand that at all times this research project will comply with the *General Data Protection Regulations (GDPR, 2018)* approved by the EU parliament on 14 April 2016 and passing into UK law effective from 25 May 2018 and that if I have any concerns how I contact the University of Leicester to raise these. I am also shared with the GDPR notice (*before filling the survey*). |  | |
| 1. I agree to take part in the above research project *follow-ups stage*. |  | |
|  | Please **initial** box | |
| 1. I agree to the use of anonymised quotes in publications. | Yes | No |
| 1. I agree that anonymised information, gathered about me for this research project may be stored in a specialist data centre/repository relevant to this subject area for future research. **The database will only be accessible to the lead researchers.** |  |  |
| 1. I agree to being named in subsequent publications and understand this will be for academic purposes and not commercial gain. |  |  |
| 1. I agree that data collected for this research project may be used in future research. |  |  |
| 1. I wish to receive a copy of the results of this research project, and I agree for my contact details to be retained and used for this purpose. |  |  |

Name of Participant Date Signature

Name of Researcher obtaining informed consent Date Signature
